# A de novo* STUB1* variant associated with an early adult-onset multisystemic ataxia phenotype

**DOI:** 10.1007/s00415-021-10524-7

**Published:** 2021-04-03

**Authors:** David Mengel, Andreas Traschütz, Selina Reich, Alejandra Leyva-Gutiérrez, Friedemann Bender, Stefan Hauser, Tobias B. Haack, Matthis Synofzik

**Affiliations:** 1 Department of Neurodegenerative Diseases, Center for Neurology, Hertie Institute for Clinical Brain Research, University of Tübingen, Tübingen, Germany; 2 German Centre for Neurodegenerative Diseases (DZNE), Tübingen, Germany; 3 Institute of Medical Genetics and Applied Genomics, University of Tübingen, Tübingen, Germany; 4 Centre for Rare Diseases, University of Tübingen, Tübingen, Germany

**Keywords:** STUB1, CHIP, Ataxia, Dominant, Early-onset ataxia, SCA48

## Abstract

**Background:**

Biallelic *STUB1* variants are a well-established cause of autosomal-recessive early-onset multisystemic ataxia (SCAR16). Evidence for *STUB1* variants causing autosomal-dominant ataxia (SCA48) so far largely relies on segregation data in larger families. Presenting the first de novo occurrence of a heterozygous *STUB1* variant, we here present additional qualitative evidence for STUB1-disease as an autosomal-dominant disorder.

**Methods:**

Whole exome sequencing on an index patient with sporadic early-onset ataxia, followed by Sanger sequencing in all family members, was used to identify causative variants as well as to rule out alternative genetic hits and intronic *STUB1* variants. *STUB1* mRNA and protein levels in PBMCs in all family members were analysed using qRT-PCR and Western Blot.

**Results:**

A previously unreported start-lost loss-of-function variant c.3G>A in the start codon of *STUB1* was identified in the index case, occurring de novo and without evidence for a second (potentially missed) variant (e.g., intronic or copy number) in *STUB1*. The patient showed an early adult-onset multisystemic ataxia complicated by spastic gait disorder, distal myoclonus and cognitive dysfunction, thus closely mirroring the systems affected in autosomal-recessive STUB1-associated disease. In line with the predicted start-lost effect of the variant, functional investigations demonstrated markedly reduced STUB1 protein expression in PBMCs, whereas mRNA levels were intact.

**Conclusion:**

De novo occurrence of the loss-of-function *STUB1* variant in our case with multisystemic ataxia provides a qualitatively additional line of evidence for STUB1-disease as an autosomal-dominant disorder, in which the same neurological systems are affected as in its autosomal-recessive counterpart. Moreover, this finding adds support for loss-of-function as a mechanism underlying autosomal-dominant STUB1-disease, thus mirroring its autosomal-recessive counterpart also in terms of the underlying mutational mechanism.

## Introduction

While variants in *STUB1* are well established to cause an autosomal-recessive early-onset multisystemic ataxia (SCAR16) [1–3], they have recently received also increasing support to be associated with autosomal-dominant ataxia (SCA48) [4–10]. Since the initial association of *STUB1* with an autosomal-dominant form of spinocerebellar ataxia (SCA48) in 2018, 34 SCA48 patients originating from 17 families have been reported [5–12]. The complex clinical phenotype invariably included ataxia and, in most cases, cognitive and/or psychiatric dysfunction, variably associated with both hyperkinetic and hypokinetic movement disorders, pyramidal tract damage, and urinary symptoms. While segregation data in multigenerational families from previous studies provide plausible evidence in support of STUB1-associated disorder as an autosomal-dominant ataxia disease, de novo occurrence of a heterozygous *STUB1* variant in an ataxia patient would contribute a strong additional qualitative line of evidence.

Here, we report a case with a de novo heterozygous start-lost c.3G>A mutation presenting with early adult-onset multisystemic ataxia complicated by cognitive dysfunction, distal myoclonus, and spastic gait disorder, thus providing qualitative novel additional evidence for STUB1-disease as an autosomal-dominant disorder. Moreover, biochemical analysis of the functional consequences of the c.3G>A *STUB1* start-lost mutation suggests loss-of-function (LoF) as an underlying mechanism in autosomal-dominant STUB1-disease.

## Methods

### Patient recruitment, assessment, and whole exome sequencing

The family (3 siblings, 2 parents, see Fig. 2b) and two unrelated controls were recruited as part of a continuous ongoing systematic deep-phenotyping and genotyping programme focussing on early-onset ataxias initiated by the Tübingen ataxia center, Hertie-Institute for Clinical Brain Research, Tübingen, Germany, since 2013. Standardized in-depth phenotyping (clinics, MRI), biosampling, and genetic work-up was performed in an index patient and, as available, his family members. A whole exome dataset with coverage on a diagnostic level (average coverage of the target region 17.6×, 96% covered > 20×) from the index patient was investigated for rare variants in known and potentially novel disease genes with strict filter sets (minor allele frequency < 0.01% in gnomAD v2.1.1, Genome Aggregation Database, gnomad.broadinstitute.org; and combined annotation dependent depletion (CADD) score (phred-like, version 1.4) > 20.

### Reagents and antibodies

All chemicals and reagents were from Millipore-Sigma (Burlington, MA, USA) unless otherwise noted.

### PBMC isolation and culture

For isolation of peripheral blood mononuclear cells (PBMCs), blood was collected into BD Mononuclear Cell Preparation Tubes (BD, Franklin Lakes, NJ, USA). Specimens were centrifuged at 1800×*g* for 20 min at room temperature (RT), the supernatant containing mononuclear cells was carefully aspirated, and cells washed using phosphate buffered saline (PBS). Residual erythrocytes were lyzed in lysis buffer (155 mM ammonium chloride, 10 mM potassium hydrogen carbonate, 0.1 mM EDTA, pH 7.4), cells were washed again in PBS, and pelleted at 300×*g* for 10 min at RT. Finally, the cell pellet was resuspended in freezing medium (FCS (Thermo Fisher, Waltham, MA, USA) + 10% DMSO) in cryotubes, and cells were frozen in liquid nitrogen until further use. PBMCs were thawed at 37 °C, resuspended in RPMI media (Thermo Fisher, Waltham, MA, USA) containing 10% FCS (Thermo Fisher, Waltham, MA, USA), and cultured for 48 h at 37 °C and 5% CO_2_.

### Generation of iPSCs and targeted STUB1 knockout with CRISPR/Cas9

The generation of iPSCs from fibroblasts was performed according to a previously published protocol [13]. In brief, human dermal fibroblasts were nucleofected with 1 µg of each episomal plasmid [pCXLE-hUL, pCXLE-hSK and pCXLE-hOCT4 (Addgene numbers 27080, 27078 and 27076, respectively)] with Nucleofector 2b (Lonza, Basel, Switzerland). Fibroblasts were cultivated in fibroblast medium before adding 2 ng/mL FGF2 (Peprotech, Hamburg, Germany) on day 2. Next day, medium was changed to Essential 8 (E8) medium with 100 μM sodium butyrate. After 3–4 weeks, colonies were manually picked and expanded onto Matrigel-coated plates (Corning, NY, USA) in a feeder-free system in E8 medium. For generation of the STUB1 knockout line *STUB1*(−/−), exons 2 and 3 of *STUB1* were targeted as previously reported [14].

### Neuronal differentiation of iPSCs

iPSC-derived neurons were generated according to a published protocol [13]. In brief, iPSCs were seeded at a density of 3 × 10^5^/cm^2^ in E8 medium supplemented with 10 µM Y-27632. The following day, the medium was replaced by neural induction medium [1:1 N2/B27, 500 nM LDN-193189 and 10 µM SB431542], which, from there onwards, was changed every day. The neural induction medium was supplemented with 20 ng/mL FGF2 on day 8. The next day, cultures were split and seeded in N2/B27 medium with 20 ng/mL FGF2 and 10 µM Y-27632 onto Matrigel-coated six-well plates. N2/B27 medium with 20 ng/mL FGF2 was added on the next day. From day 11 onward, cells were cultured in N2/B27 medium with a change of medium every other day. On day 26, cells were detached with Accutase and reseeded at 4 × 10^5^/cm^2^ on poly-l-ornithine and Matrigel-coated wells. On day 27 and 29, the medium was changed to N2/B27 supplemented with 10 µM PD0325901 (Tocris, Bristol, UK) and 10 µM DAPT. From day 31 on cells were cultured in N2/B27 medium until day 36 on which cells harvested for protein isolation.

### Protein isolation

PBMCs and iPSC-derived neurons were lysed in RIPA buffer containing 1× complete protease inhibitor cocktail (Roche, Mannheim, Germany) for 30 min on ice, and vortexed every 10 min for 30 s. Cell debris was pelleted at 20,000×*g* for 15 min at 4 °C. Protein concentration was determined using a BCA protein assay (Thermo Fisher Scientific, Waltham, MA, USA) according to the manufacturer's instructions.

### Western blot analysis

Proteins were electrophoresed on hand-cast 10% Bis–Tris gels and transferred onto 0.45 μm PVDF membrane (Merck-Millipore, Burlington, MA, USA) at 100 V for 2 h. Blots were incubated in tris-buffered saline containing 0.1% Tween-20 (TBS-T) and 5% non-fat dry milk for 1 h at RT, and then incubated with primary antibody (0.1 μg/mL anti-STUB1, ab134064, Abcam, Cambridge, UK) in 1 × Roche Block (Roche Diagnostics, Mannheim, Germany) overnight at 4 °C. Blots were then washed three times with TBS-T for 10 min. Membranes were incubated with horseradish peroxidase-conjugated anti-rabbit antibodies (Jackson Immunosearch, Westgrove, PA, USA) for 1 h at RT, followed by 3 × 10 min washing with TBS-T. Immunoreactive bands were detected using ECL (Immobilon Western HRP Substrat, Merck-Millipore, Burlington, MA, USA) and visualized using the ChemiDOC MP Imaging System (Bio-Rad, Hercules, CA, USA). Next, blots were stripped with stripping buffer (0.025 M Glycine, 1% SDS, 1% Tween-20, pH 2.0) for 10 min, washed 3 × 10 min with TBS-T, and then reprobed with anti-β-actin monoclonal antibodies (0.03 μg/mL in 1 × Roche Block, Merck-Millipore, Burlington, MA, USA) as loading control. Bands were quantified with ImageJ and normalized to the loading control.

### Quantitative real-time PCR

Total mRNA was isolated from PBMCs using the High Pure RNA Isolation Kit (Roche Diagnostics, Mannheim, Germany) according to the manufacturer´s protocol. Concentration and quality of RNA was determined by measuring absorbance at 260/280 nm with a NanoDrop ND1000 spectrophotometer (VWR, Radnor, PA, USA). A total of 200 ng RNA was reverse-transcribed into cDNA using random hexamer primers and the Transcriptor High Fidelity cDNA Synthesis Kit (Roche Diagnostics, Mannheim, Germany) according to the manufacturer's instructions. For mRNA expression analysis, cDNA was diluted 1:10 and quantitative real-time PCR was performed on a Light Cycler 480 (Roche Diagnostics, Mannheim, Germany) using a touchdown PCR protocol. 1.5 µL of cDNA dilution was mixed with 1.5 µL of 20 µM primer mix (forward: TCAAGGAGCAGGGCAATCGT; reverse: CAGCGGGTTCCGGGTGAT) and 7.5 µL of LightCycler^®^ 480 SYBR Green I master mix (Roche Diagnostics, Mannheim, Germany). The specificity of PCR products was confirmed by melting curve analysis and efficiency was determined using standard curves. Housekeeping genes *B2M, RNF111* and *RNF10* were amplified to standardize the amount of sample cDNA. Analysis was performed with advanced relative quantification on the Light Cycler 480 Software 1.5.1.62 (Roche Diagnostics, Mannheim, Germany).

## Results

**Fig. 1:**
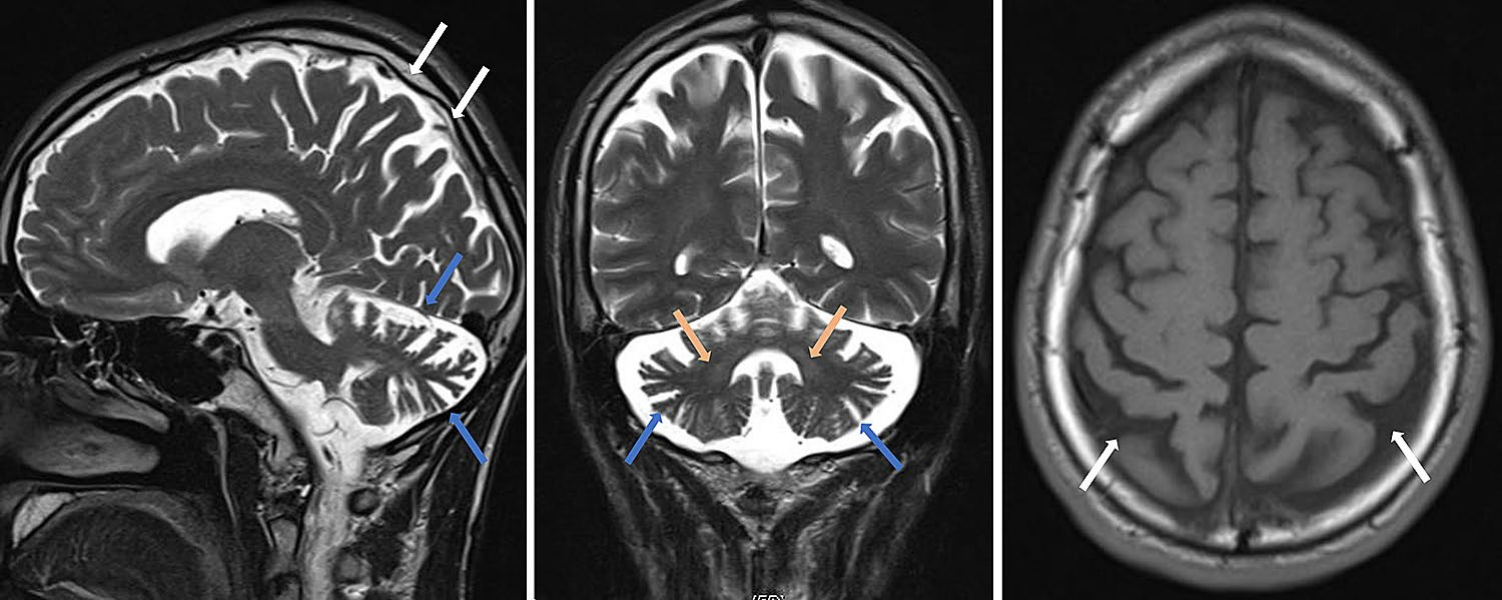
**Imaging findings of the index patient with the heterozygous de novo *STUB1*** **variant**. T2-weighted sagittal (left) and coronal (middle) and T1-weighted axial (right) MRI imaging showing severe cerebellar degeneration (blue arrows), atrophy of the parietal lobes (white arrows) and hyperintensity of both dentate nuclei (dark yellow arrows)

**Fig. 2:**
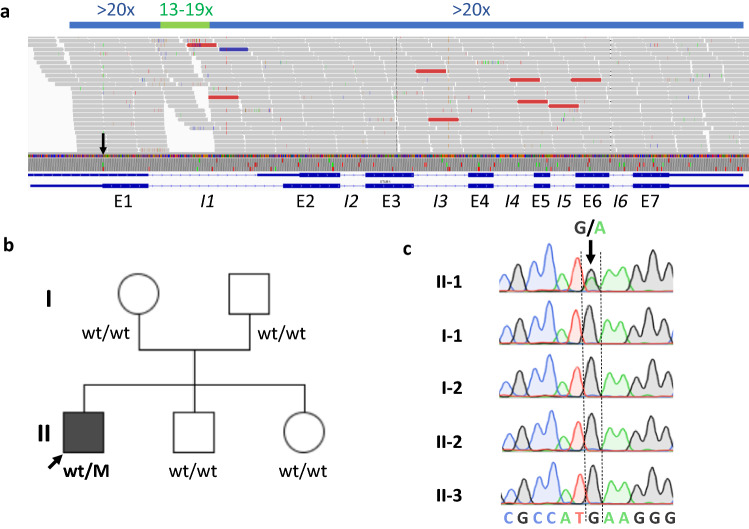
**Genetic findings of the index patient and his family.** **a** WES of the index patient shows that all exonic (E1–E7) and intronic regions (*I1*–*I6*) were covered with a coverage of ≥ 20× (except a 56 bp region in intron 1, which was however, also still covered with ≥ 13×, indicated in light green). The heterozygous c.3G > A *STUB1* variant in the initiation codon is indicated with a black arrow. **b** Pedigree tree and **c** electropherograms demonstrating de novo occurrence of the heterozygous c.3G>A *STUB1* variant (= M) in the index patient. This mutation was absent in both unaffected parents and siblings. The filled symbol represents the affected patient, and open symbols asymptomatic family members. The index patient is marked with an arrow

A 34-year-old man from non-consanguineous German parents without family history of ataxia or dementia presented with progressive upper limb hyperkinetic movements starting at age 30 years, subtle and slowly progressive gait disturbance starting at age 33 years, and impaired fine motor skills starting age 34 years. Childhood development had been normal and after finishing high school, he had worked as a medical technician. Upon neurological examination, the patient showed—apart from cerebellar oculomotor disturbances (saccadic smooth pursuit eye movements, hypermetric saccades), truncal (ataxic stance and gait) and appendicular ataxia (limb dysmetria, kinetic tremor)—myoclonic jerks of the upper limbs, and spastic gait disturbance. Chorea, dystonia, parkinsonism, epilepsy, and urinary tract symptoms were absent. Clinical signs of neuropathy were not present, and secondary sexual characteristics were normal. In the consecutive 3 years from first examination, gait and stance became more unstable and upper limb ataxia worsened, as reflected by an increase of 2.5 points on the Scale for the Assessment and Rating of Ataxia (SARA: 15to 17.5 points) [15]. Neuropsychological evaluation including the Test battery for attentional performance (TAP), the Wechsler Memory Scale-Revised (WMS-R) and the Verbal learning and memory test (VLMT) indicated pronounced attention deficits and a decline in memory performance (age and education adjusted scores were lower or far below average). Visuospatial dysfunction, affective and behavioural symptoms were not observed, thus not meeting the full characteristics of the cerebellar cognitive affective syndrome [16], as described for other previous STUB1 patients [4, 6–8]. Due to cognitive decline and worsening ataxia, he had to change his workplace within the company and to reduce the workload to part-time. Prolongation of the central motor conduction time to the lower limbs provided corroborative electrophysiological evidence for pyramidal tract damage. Nerve conduction studies revealed—clinically inapparent—axonal motor neuropathy. In addition to severe cerebellar atrophy, MRI brain scan showed T2-weighted hyperintensity in the dentate nuclei and bilateral parietal atrophy (Fig. 1). Whole-exome sequencing of the index patient revealed a previously unreported heterozygous start-lost loss-of-function (LoF) variant c.3G>A in the start codon of *STUB1* (NM_005861.4: p.?). This variant is absent in both 13,140 in-house WES/WGS datasets as well as in the 276,000 alleles of gnomAD. Not only all exonic, but also all intronic regions were covered with a coverage of > 20× (except merely a 56 base pair region in intron 1, which was still covered with > 13×) by whole exome sequencing, making it unlikely that a putative second variant in the intronic region of *STUB1* (and thus a possible autosomal-recessive mode of inheritance) would have been missed (Fig. 2a). Likewise, WES-based CNV analysis by ClinCNV (https://www.biorxiv.org/content/10.1101/837971v1) did not reveal any CNVs within the chromosomal region 16p13.3. Together with the identification of two SNPs in neighbouring *RHBDL1* in a heterozygous state (rs111852492 and rs370469600, located 3369 and 2851 bp upstream of the *STUB1* transcript, respectively), these analyses did not provide any evidence that a putative CNV (e.g., a microdeletion) would have been missed. Sanger sequencing confirmed presence of the variant in the index patient, and its absence in the other family members including both parents (Fig. 2b, c), with paternity confirmed by analysis of five short-tandem-repeat (STR) loci in the index patients and both parents, thus demonstrating de novo occurrence of this variant. The absence of the variant not only in the parents, but also in both siblings (Fig. 2b) not only corroborates potential pathogenicity of the variant, but also makes it less likely that a putative mosaicism in the parents might have been overlooked.

**Fig. 3:**
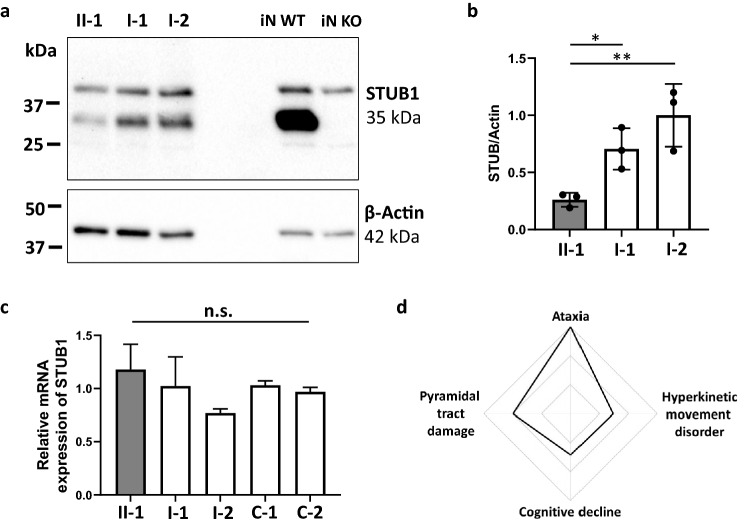
**STUB1 protein and mRNA expression in PBMCs, and phenotypic spectrum of de novo** **STUB1-disease in the index patient**. mRNA and proteins were extracted from PBMCs of the index patient (II.1) and his parents (I.1 and I.2). **a** Levels of STUB1 protein were quantified using Western Blot analysis, and were markedly reduced in the index patient (II.1) compared to both parents (I.1 and I.2). Equal protein loading is demonstrated using anti-β-actin monoclonal antibodies. Specificity of the antibodies was demonstrated using cell lysates from iPSC-derived neurons. A strong band was visible at the predicted molecular weight of 35 kDa for STUB1 wilde-type (iN WT), but not STUB1 knock-out cells (iN KO). **b** Densitometric analysis of three independent experiments shows decreased STUB1 expression in the index patient relative to β-actin. **c** Expression of *STUB1* mRNA was measured using qRT-PCR (in three independent experiments), and was similar in the index patient and his parents, and two unrelated controls (C-1 and C-2). Differences in means for **b **and **c **were assessed with ANOVA followed by Tukey’s *post-hoc* test. Means and standard deviations are shown. n.s., non-significant (*p* > 0.05), *p < 0.05, **p < 0.01. **d** The phenotypic spectrum of the index patient with de novo STUB1-disease consists of ataxia, pyramidal tract damage, hyperkinetic movement disorder, and cognitive decline as part of the multisystemic disease spectrum in STUB1-disease

The c.3G>A variant was predicted in silico to cause a start-lost effect on protein translation. In line with this prediction, STUB1 protein expression in PBMCs was markedly decreased in the mutation carrier (reduced to ~ 30% compared to his parents; Fig. 3a, b). Specificity of the antibody to detect STUB1 was demonstrated by use of cell lysates of STUB1 iPSC-derived neuronal cells where a strong band was visible at the predicted molecular weight of 35 kDa for STUB1 wild-type, but not STUB1 knock-out cells, serving as positive and negative controls, respectively, for the Western Blot analyses. In sum, these protein analyses suggest loss-of-function (LoF) as the underlying disease mechanism of this *STUB1* variant.

As predicted for a start-lost variant that only affects protein translation, not transcription, mRNA levels encoded by *STUB1* were unchanged in PBMCs (Fig. 3c). Both STUB1 protein expression and mRNA levels were normal in the parents (Fig. 3a–c), adding further evidence that no putative mosaicism or second, inherited *STUB1* variant (e.g., intronic or CNV) in the family has been missed.

## Discussion

With the first report of a de novo occurrence of a heterozygous *STUB1* variant, our study adds a qualitative novel line of evidence that *STUB1* variants, especially LoF variants, indeed cause also *autosomal-dominant* ataxia (SCA48). We functionally confirmed the predicted start-lost effect of the variant, and ruled out other modes of inheritance other than de novo occurrence. Specifically, no evidence was found that this *STUB1* variant was putatively *inherited* (and present e.g., just as a mosaic in one of the parents), with absence of the variant in all family members, including two siblings, and normal *STUB1* mRNA and protein expression levels in the parents. No evidence was found for a potential *autosomal-recessive* state of the variant, with a putative second intronic or structural *STUB1* variant in the index patient ruled out by whole exome sequencing. This finding of de novo occurrence of *STUB1* disease corroborates and extends current evidence of STUB1-disease as an autosomal-dominat disorder, which so far has relied mainly on segregation data in larger families and enrichment in mutational burden analyses [4, 5].

Moreover, our findings suggest loss-of-function (LoF) as an underlying mechanism in autosomal-dominant STUB1-disease, at least for the *STUB1* mutation investigated here. These findings nicely complement findings from heterozygous STUB1 −/+ mice, which suggested haploinsufficiency as a possible disease mechanism in murine autosomal-dominant STUB1-disease [17]. Moreover, they mirror clinical and experimental studies from autosomal-recessive STUB1-disease, where LoF has also been suggested as a causative mechanism [5]. However, STUB1 gain-of-function and dominant negative effects have also been discussed for autosomal-dominant STUB1-disease [5, 7, 10]. Additional functional studies are warranted to better understand the mutational disease mechanisms underlying autosomal-dominant STUB1-disease, ideally by a comparative parallel investigation of multiple *STUB1* mutations of different variant types (e.g., missense, splice, stop), and from different protein domains.

Our findings thus not only provide corroborative *genetic* and preliminary *mechanistic* evidence for STUB1-disease as an autosomal-dominant disorder, but also add further *phenotypic* evidence for the similarity of autosomal-dominant STUB1 disease with its autosomal-recessive counterpart. Autosomal-recessive STUB1-disease has been described to cause an early-onset multisystemic ataxia including cerebellar ataxia, pyramidal tract damage, hyperkinetic movement disorders and cognitive decline as the main affected systems [1, 2]. These systems—as well as the early-onset of the disease—are exactly all mirrored by the case presented here, which presented with cerebellar ataxia, distal myoclonus, spastic gait disorder and cognitive dysfunction (Fig. 3d), and with structural brain damage extending beyond the cerebellum to the cerebral cortex. The presence of bilateral signal alterations of the dentate nuclei along with cerebellar atrophy support the notion of a common neuroradiological feature in SCA48, recently referred to as “crab sign” due to its morphological appearance [18]. Although this striking MRI imaging sign is very rare in hereditary ataxias, systematic imaging studies are warranted to investigate whether it is really fully specific to STUB1-disease.

Taken together, our findings (1) add an additional qualitative line of evidence for the existence of autosomal-dominant STUB1-disease (hereby corroborating several recent studies [6, 9]), (2) provide support for loss-of-function as a mechanism underlying autosomal-dominant STUB1-disease, thus mirroring its autosomal-recessive counterpart and (3) demonstrate that autosomal-dominant STUB1-disease affects the same neurological systems as its autosomal-recessive counterpart. This suggests, in sum, that both modes of inheritance can be associated with a similar STUB1 disease condition and possibly even similar mutational mechanism.

## Data Availability

Not applicable.
